# Correction: mTORC1 in the orbitofrontal cortex promotes habitual alcohol seeking

**DOI:** 10.7554/eLife.79782

**Published:** 2022-05-04

**Authors:** Nadege Morisot, Khanhky Phamluong, Yann Ehinger, Anthony L Berger, Jeffrey J Moffat, Dorit Ron

**Keywords:** Rat

Morisot N, Phamluong K, Ehinger Y, Berger AL, Moffat JF, Ron D. 2019. mTORC1 in the orbitofrontal cortex promotes habitual alcohol seeking. *eLife* **8**:e51333. doi: 10.7554/eLife.51333.

We identified an error in Figure 5e of this manuscript. Specifically, Figure 5e depicts the effect of intra-orbitofrontal cortex (OFC) administration of the NMDA receptor antagonist, Ro25 on habitual alcohol lever presses. We discovered that confirmation of cannula placement was not performed for an entire cohort of rats used for this study. We recognized this issue before submitting the manuscript and intended to remove all the rats that were missing canulae placement from the Figure but made a mistake in this process, which led to the removal of rats that should have remained and the inclusion of rats that should have been excluded from the report.

Correcting the error outlined above reduced the number of animals we could analyse for this panel (from n = 12 to n = 6), and results in a corresponding change in p-value for the Sidak’s multiple comparison between non-devaluation (ND) and devaluation (D) days (from p < 0.01 to p < 0.05) in Ro25-treated rats. In addition, with the smaller group size, the main effect of devaluation and the treatment x devaluation interaction are no longer statistically significant. However, post-hoc power analysis using G*Power software (Heinrich Heine University Düsseldorf), incorporating the observed effect size and updated group sizes, indicates that the corrected analysis is still sufficiently powered, according to our initial standards [Power (1 – β err prob) > 0.8].

Although the conclusion from the experiment depicted in Figure 5e, that GluN2B in the OFC plays a role in habitual alcohol seeking, remains unchanged, we adjusted the text to reflect the comparably lower statistical power in the corrected analysis. We sincerely apologize for this oversight.

We updated the manuscript and Figure 5e to reflect these corrections and outlined them below:

1. Original and corrected sections of the text are detailed below, with revised text in bold.

Original:

- In contrast, administration of Ro25-6981 (5 μg/μl) into the OFC reverted habitual responding for alcohol to goal-directed responding (Figure 5e, Source data Figure 5). Together, these data indicate that the recruitment of GluN2B signalling by alcohol is required for alcohol seeking and habit.
- We show here that GluN2B activation in the OFC is sufficient to influence the formation and/or maintenance of alcohol seeking and habit.

Corrected:

- In contrast, administration of Ro25-6981 (5 μg/μl) into the OFC **shifts habitually** responding **rats to be more** goal-directed **in their** responding **for alcohol** (Figure 5e, Source data Figure 5). Together, these data indicate that the recruitment of GluN2B signaling by alcohol **may be involved in driving** alcohol seeking and habit.
- We show here that GluN2B activation in the OFC **may be** sufficient to influence the formation and/or maintenance of alcohol seeking and habit.

2. Original and corrected portion of the legend for Figure 5 with changes in bold:

Original:

- (e) Intra-OFC administration of Ro25-6981 attenuates habitual alcohol seeking. Two way RM ANOVA showed a main effect of devaluation (F_1,33_=13.72, p < 0.001) as well as a treatment x devaluation interaction (F_1,33_=6.31, p<0.05). Sidak’s multiple comparison test detected a significant difference for Ro25-6981 (p<0.001) on D compared to ND days. Individual data points and mean ± SEM are shown, (a–c) n = 7, (e) n = 12. *p<0.05, **p< 0.01.

Corrected:

- (e) Intra-OFC administration of Ro25-6981 attenuates habitual alcohol seeking. Two way RM ANOVA **did not detect** a main effect of devaluation (**F_1,5_=4.162, p=0.0969**) **or** a treatment x devaluation interaction (**F_1,5_=1.956, p=0.2208**). Sidak’s multiple comparison test detected a significant difference for Ro25-6981 (p**<0.05**) on D compared to ND days. Individual data points and mean ± SEM are shown, (a–c) n = 7, (e) n= **6**. *p<0.05.

3. Original and corrected Figure 5e:

Original:

**Figure 1:**
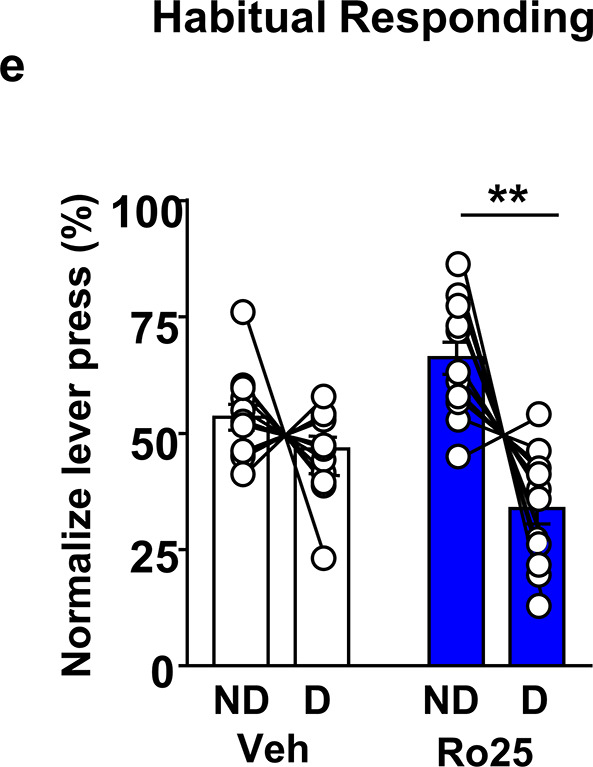


Corrected:

**Figure 2:**
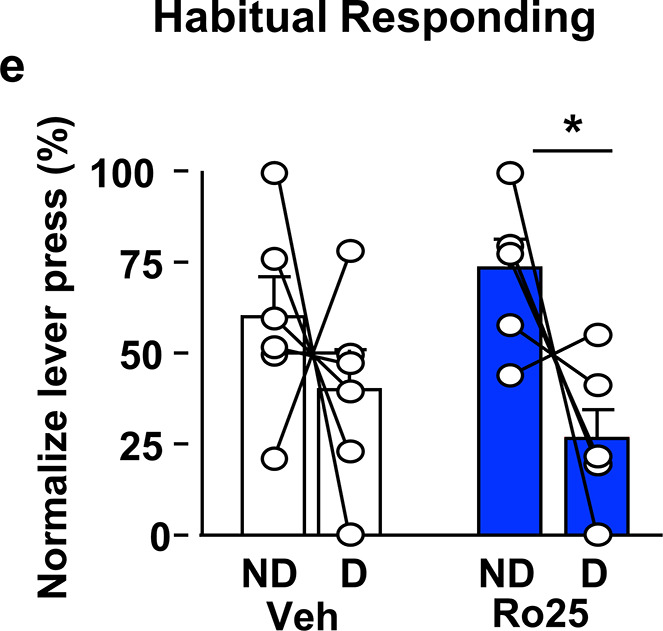


4. Original and corrected portion of Table 1:

Original:

**Table:** 

Alcohol Devaluation							
	Training	RI		RR		RI	
	Group	Vehicle	rapamycin	Vehicle	rapamycin	Vehicle	Ro25
Non-devalued day	1% Sucrose (ml/kg)	1.4	2.0	1.2	1.9	2.7	4.5
		±	±	±	±	±	±
		0.5	0.8	0.4	0.6	1.5	0.9
	Water (ml/kg)	0.7	0.6	1.2	0.8	1.0	2.5
		±	±	±	±	±	±
		0.3	0.1	0.7	0.1	0.2	1.2
Devalued day	20% Alcohol (g/kg)	0.7	0.7	1.0	0.7	0.9	1.0
		±	±	±	±	±	±
		0.1	0.1	0.1	0.1	0.1	0.2
	Water (ml/kg)	0.4	0.6	0.7	0.6	1.0	0.9
		±	±	±	±	±	±
		0.1	0.1	0.1	0.1	0.4	0.2

Corrected:

**Table:** 

Alcohol Devaluation							
	Training	RI		RR		RI	
	Group	Vehicle	rapamycin	Vehicle	rapamycin	Vehicle	Ro25
Non-devalued day	1% Sucrose (ml/kg)	1.4	2.0	1.2	1.9	**2.7**	**5.8**
		±	±	±	±	±	±
		0.5	0.8	0.4	0.6	**1.5**	**1.5**
	Water (ml/kg)	0.7	0.6	1.2	0.8	**1.0**	**1.3**
		±	±	±	±	±	±
		0.3	0.1	0.7	0.1	**0.2**	**0.3**
Devalued day	20% Alcohol (g/kg)	0.7	0.7	1.0	0.7	**1.0**	**1.3**
		±	±	±	±	±	±
		0.1	0.1	0.1	0.1	**0.1**	**0.1**
	Water (ml/kg)	0.4	0.6	0.7	0.6	**1.0**	**0.9**
		±	±	±	±	±	±
		0.1	0.1	0.1	0.1	**0.4**	**0.2**

5. Figure 5—source data 3 was also replaced.

The article has been corrected accordingly.

