# Supplementary figures and images for: mTORC1 in the orbitofrontal cortex promotes habitual alcohol seeking
Source: eLife. 2019 Dec 11;8:e51333. doi: 10.7554/eLife.51333 (PMC6959998; doi:10.7554/eLife.51333)

## Slide 1
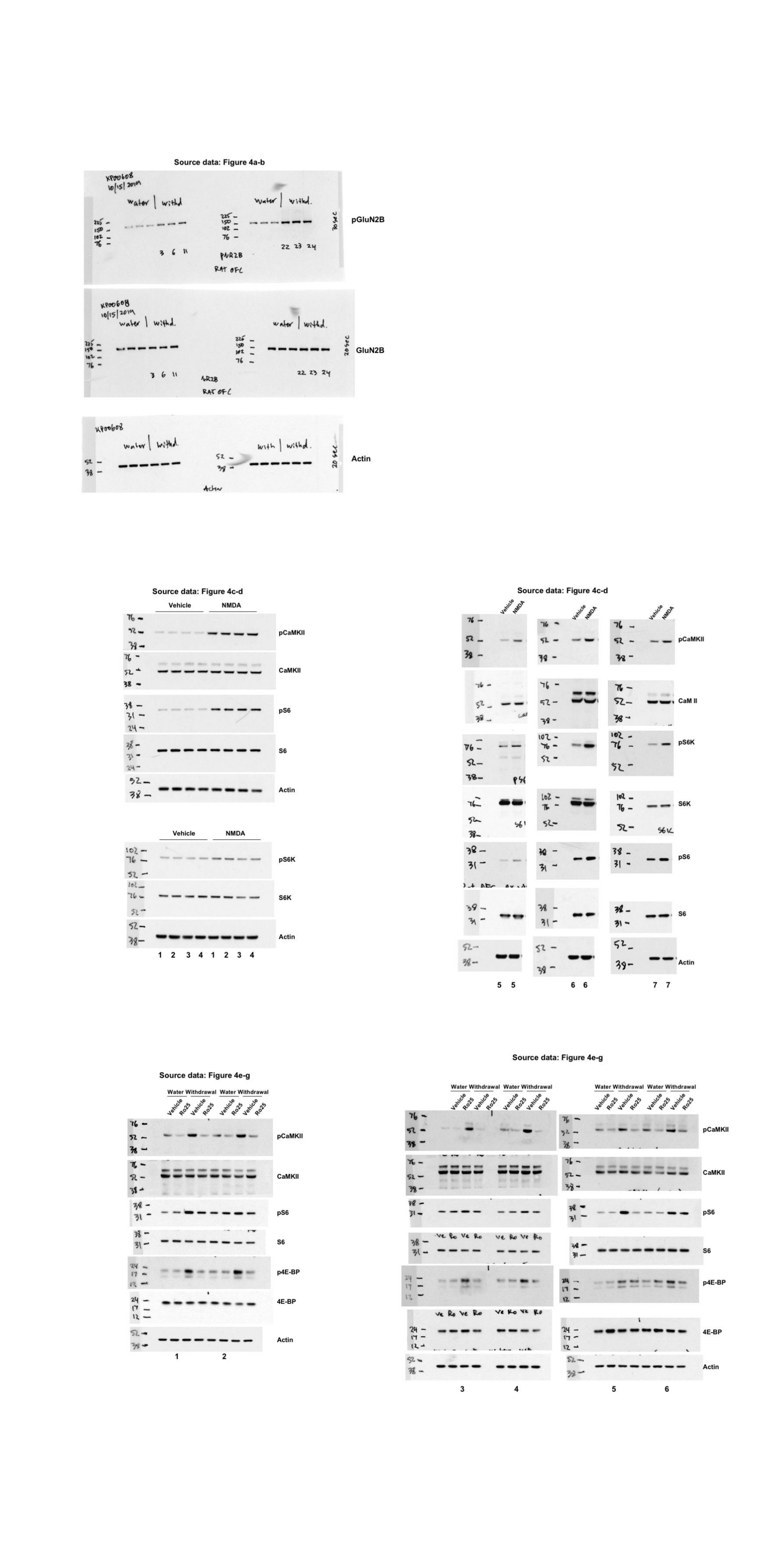

Supplement: Figure 4—source data 1. [file elife-51333-fig4-data1.pptx]
